# Skeletal diseases caused by mutations in *PTH1R* show aberrant differentiation of skeletal progenitors due to dysregulation of DEPTOR

**DOI:** 10.3389/fcell.2022.963389

**Published:** 2023-01-16

**Authors:** Fabiana Csukasi, Michaela Bosakova, Tomas Barta, Jorge H. Martin, Jesus Arcedo, Maya Barad, Gustavo A. Rico-Llanos, Jennifer Zieba, Jose Becerra, Pavel Krejci, Ivan Duran, Deborah Krakow

**Affiliations:** ^1^ Department of Orthopaedic Surgery, David Geffen School of Medicine at University of California at Los Angeles, Los Angeles, CA, United States; ^2^ Laboratory of Bioengineering and Tissue Regeneration (LABRET), Department of Cell Biology, Genetics and Physiology, University of Malaga, Institute of Biomedical Research in Malaga (IBIMA-Plataforma BIONAND), Malaga, Spain; ^3^ Biomedical Research Networking Center in Bioengineering, Biomaterials, and Nanomedicine (CIBER-BBN), Madrid, Spain; ^4^ Department of Biology, Faculty of Medicine, Masaryk University, Brno, Czechia; ^5^ Institute of Animal Physiology and Genetics of the CAS, Brno, Czechia; ^6^ International Clinical Research Center, St. Anne’s University Hospital, Brno, Czechia; ^7^ Department of Histology and Embryology, Faculty of Medicine, Masaryk University, Brno, Czechia; ^8^ Department of Human Genetics, David Geffen School of Medicine at University of California at Los Angeles, Los Angeles, CA, United States; ^9^ Department of Obstetrics and Gynecology, David Geffen School of Medicine at University of California at Los Angeles, Los Angeles, CA, United States; ^10^ Department of Pediatrics, David Geffen School of Medicine at University of California at Los Angeles, Los Angeles, CA, United states

**Keywords:** DEPTOR, TAZ, osteogenesis, PTH signaling, Wnt, skeletal differentiation

## Abstract

Alterations in the balance between skeletogenesis and adipogenesis is a pathogenic feature in multiple skeletal disorders. Clinically, enhanced bone marrow adiposity in bones impairs mobility and increases fracture risk, reducing the quality of life of patients. The molecular mechanism that underlies the balance between skeletogenesis and adipogenesis is not completely understood but alterations in skeletal progenitor cells’ differentiation pathway plays a key role. We recently demonstrated that parathyroid hormone (PTH)/PTH-related peptide (PTHrP) control the levels of DEPTOR, an inhibitor of the mechanistic target of rapamycin (mTOR), and that DEPTOR levels are altered in different skeletal diseases. Here, we show that mutations in the PTH receptor-1 (PTH1R) alter the differentiation of skeletal progenitors in two different skeletal genetic disorders and lead to accumulation of fat or cartilage in bones. Mechanistically, DEPTOR controls the subcellular localization of TAZ (transcriptional co-activator with a PDZ-binding domain), a transcriptional regulator that governs skeletal stem cells differentiation into either bone and fat. We show that DEPTOR regulation of TAZ localization is achieved through the control of Dishevelled2 (DVL2) phosphorylation. Depending on nutrient availability, DEPTOR directly interacts with PTH1R to regulate PTH/PTHrP signaling or it forms a complex with TAZ, to prevent its translocation to the nucleus and therefore inhibit its transcriptional activity. Our data point DEPTOR as a key molecule in skeletal progenitor differentiation; its dysregulation under pathologic conditions results in aberrant bone/fat balance.

## Introduction

Skeletal progenitors are found in the bone marrow (BM) and give rise to cartilage, bone and fat through different temporal cell fate choices. During embryogenesis and post embryonic development, cartilage forms first, then bone, and adipocytes arise at post embryonic stages, accumulating steadily throughout the lifespan (Chan et al., 2015; Ambrosi et al., 2017; Chan et al., 2018; Yu et al., 2018). The differentiation of these skeletal progenitors is a tightly regulated process and an altered balance between skeletogenesis and adipogenesis underlies numerous disease pathologies. Enhanced BM adiposity is caused by genetic and environmental factors that determine a switch from the osteogenic to the adipogenic lineage, leading to decreased bone density and increased fragility (Devlin and Rosen, 2015; Veldhuis-Vlug and Rosen, 2017).

Developmental commitment to chondrogenic, osteogenic, and adipogenic lineages is regulated by three master transcription factors: SRY-box containing gene 9 (SOX9) for chondrogenesis; runt-related transcription factor 2 (RUNX2), for osteogenesis, and peroxisome proliferator-activated receptor (PPARγ), for adipogenesis (Kozhemyakina et al., 2015). Multiple signaling pathways are involved in skeletal progenitors differentiation including bone morphogenetic proteins (BMPs), hedgehog (Hh), and wingless-related integrator site (WNT) (Cook and Genever, 2013). One less studied but essential pathway mediating skeletal lineage commitment is parathyroid hormone (PTH) and its related protein, PTH-related protein (PTHrP). Recent studies suggest that PTH can recruit cellular progenitors into the osteoblast lineage, resulting in increased bone formation (Yu et al., 2012a), while the PTH receptor-1 (PTH1R) ligand, PTHrP, has been shown to inhibit adipocyte differentiation (Chan et al., 2001). Mice heterozygous for inactivation of the *Pthrp* gene exhibited low bone mass and increased marrow adiposity (Amizuka et al., 1996). Transgenic mice expressing constitutively activated *Pthr1* showed delayed skeleton mineralization, delayed conversion of proliferative chondrocytes into hypertrophic cells and prolonged presence of hypertrophic chondrocytes with delay of vascular invasion (Schipani et al., 1997). Conditional deletion of PTH1R in mice using *Prx-Cre*, a limb progenitor model, also resulted in a substantial increase in BM fat, accompanied by high bone resorption (Fan et al., 2017). These varying effects of PTH/PTHrP activity are seen in two skeletal dysplasias that result from mutations in *PTH1R*; Jansen Metaphyseal Chondrodysplasia (JMC) (Cohen, 2002), an autosomal dominant disease that results from constitutive action of PTH1R (Schipani et al., 1995) and Blomstrand Chondrodysplasia (BOCD) (Loshkajian et al., 1997), an autosomal recessive disorder caused by loss-of-function mutations in *PTH1R* (Jobert et al., 1998). JMC shows delayed chondrocyte differentiation and bone formation, and conversely, BOCD patients show advanced bone maturation (Jobert et al., 1998; Kozlowski et al., 1999).

In the past few years, the role played by the mechanistic target of rapamycin (mTOR) in skeletal development has been address by several groups (Oh et al., 2001; Phornphutkul et al., 2008; Chen and Long, 2014; Chen et al., 2015; Fang et al., 2015; Huang et al., 2015; Dai et al., 2016; Yan et al., 2016; Jiang et al., 2017; Zhang et al., 2017). In particular, one member of the mTOR complex 1 (mTORC1) and mTOR complex 2 (mTORC2) that has recently gained considerable attention is the DEP-domain containing mTOR-interacting protein (DEPTOR). DEPTOR was identified as a negative regulator of mTORC1/2 complexes but its mechanism of action is poorly understood (Peterson et al., 2009). We previously reported aberrant levels of DEPTOR in two different skeletal genetic disorders: JMC and spondyloepimethapyseal dysplasia, Krakow type, and demonstrated that decreased mTORC1/2 activity due to accumulation of DEPTOR contributed to the skeletal defects (Csukasi et al., 2018). We showed that the increased levels of DEPTOR resulted in a severe reduction in the number of hypertrophic chondrocytes (Csukasi et al., 2018). Another work linking DEPTOR with growth plate cartilage demonstrated a connection between DEPTOR and the development of osteoarthritis (Li et al., 2021). Low bone mass and vertebral stiffness were reported in DEPTOR knockout mice due to dysregulation in bone resorption (Pereira et al., 2020). High levels of DEPTOR have also been correlated to osteoporosis progression, and DEPTOR is proposed to be an inhibitor of osteogenic differentiation in BM mesenchymal stem cells (BMSC) (Chen et al., 2018).

Here, we describe a dysregulation in the differentiation of skeletal progenitors in two distinct disorders with mutations in the gene encoding *PTH1R*, JMC and BOCD, which lead to accumulation of fat and invasion of chondrocytes into bone respectively. We describe a mechanism of disease involving DEPTOR control of the subcellular localization of TAZ (transcriptional co-activator with a PDZ-binding domain), a transcriptional modulator of skeletal stem cells differentiation. Our results suggest that DEPTOR acts as a molecular switch between osteogenesis and adipogenesis under pathological conditions.

## Results

### Activating and loss-of-function mutations in PTH1R result in aberrant skeletal progenitor differentiation

**FIGURE 1:**
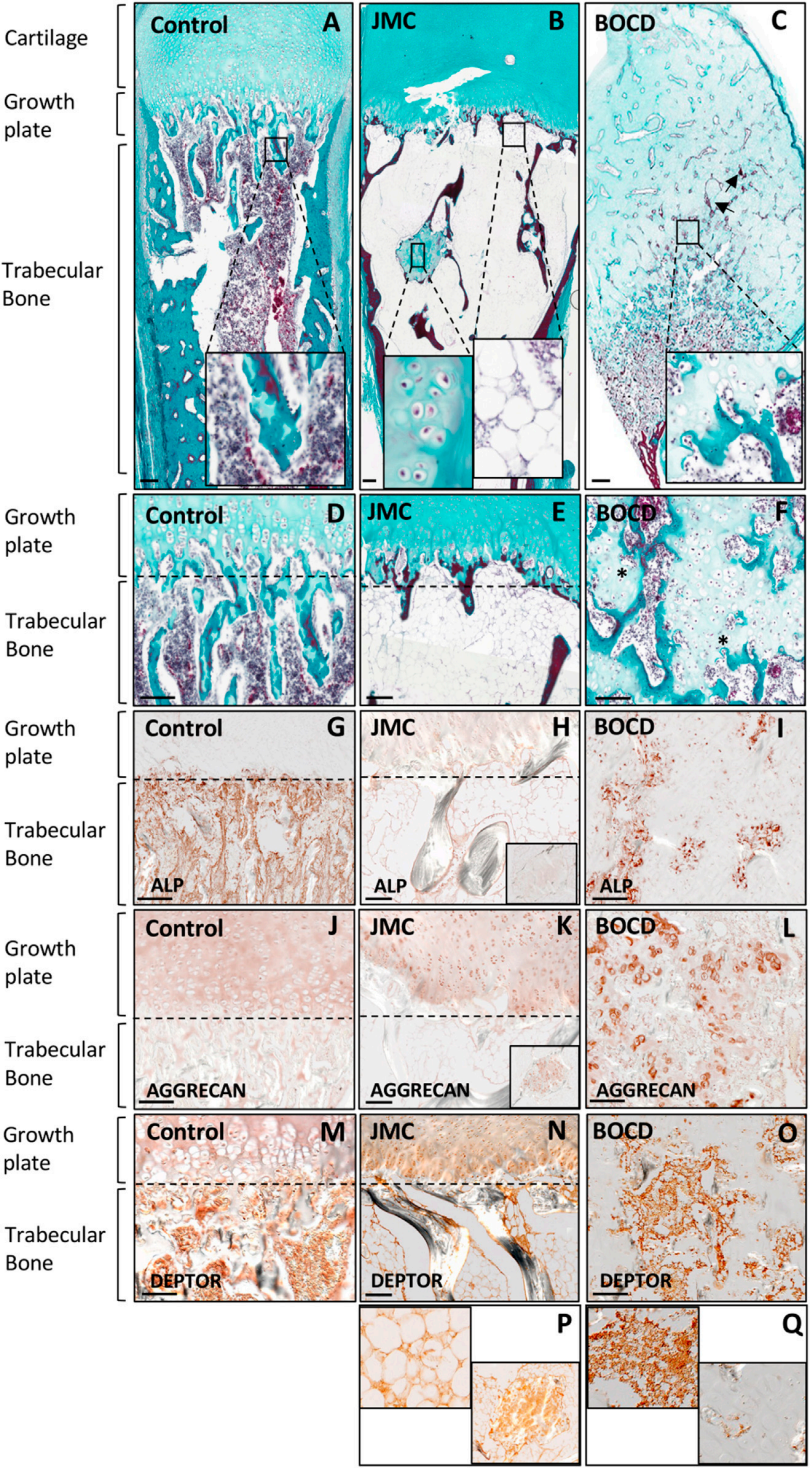
JMC and BOCD patients show aberrant differentiation of skeletal progenitors. **(A–F)** Masson Goldners staining of Control **(A, D)**, JMC **(B, E)** and BOCD **(C, F)**. Note the accumulation of fat and the small region of cartilage in the trabecular bone of JMC patients (magnification in B), and the invasion of chondrocytes in the trabecular bone of BOCD patients (magnification in C). Arrows point to calcified islands, asterisks indicate undermineralized trabecular bone. **(G–I)** Immunolocalization of ALP of Control **(G)**, JMC **(H)** and BOCD **(I)**. (J–L) Immunolocalization of AGGRECAN in Control **(J)**, JMC **(K)** and BOCD **(L)**. ALP staining demonstrates decreased bone mass in JMC and BOCD patients; AGGRECAN immunolocalization confirms the presence of cartilage in JMC and BOCD trabecular bone. **(M–Q)** Immunolocalization of DEPTOR in Control **(M)**, JMC **(N, P)** and BOCD **(O, Q)**. JMC, Jansen Methaphyseal Chondrodysplasia; BOCD, Blomstrand Chondrodysplasia; ALP, Alkaline phosphatase. Scale bars, 100 μm.

JMC and BOCD are caused by activating and loss-of-function mutations in the PTH/PTHrP receptor (PTH1R), respectively. We previously demonstrated that JMC patients show dysregulation of cartilage differentiation (Csukasi et al., 2018). To determine if the mutations in PTH1R also affect bone formation we performed histological analyses of JMC and BOCD long bones. Compared to control bone, JMC bone showed a reduced number of trabecular bone structures (Figures 1A, B), which was replaced by BM markedly enriched with fat (Figure 1B insert, E). Within the marrow cavity, JMC bone also presented unusual cartilage islands fully surrounded by fat (Figure 1B insert, E). Immunohistochemistry (IHC) analyses confirmed the reduced osteogenic differentiation of JMC trabecular bone as observed by alkaline phosphatase (ALP) staining (Figures 1G, H), while expression of the chondrocyte protein (Aggrecan) confirmed the chondrogenic nature of the ectopic cartilaginous islands within the diaphyseal cavity (Figure 1K insert). Histologic analysis of BOCD bone showed (Figure 1C) a very disorganized growth plate with no distinguishable layered organization of -resting-proliferating-hypertrophic-chondrocytes (Figures 1C, F). BOCD histology also revealed invading ectopic calcified islands within the hypertrophic zone (arrows in C) and undermineralized and disordered trabecular bone (asterisks in F). Alkaline phosphatase (ALP) staining confirmed the irregular ossification in BOCD trabeculae (Figure 1I), while the chondrocyte marker (AGGRECAN) revealed the presence of ectopic chondrocytes within the trabecular-like structures of BOCD diaphysis (Figure 1L). Altogether, these results demonstrate significant irregularities in the organization of the cartilage growth plate and bone, suggesting that cell fate decisions may be altered due to mutations in the gene that encodes PTHR1.

Our previous work demonstrated that JMC derived cells and cartilage growth plate had accumulation of the mTOR inhibitor DEPTOR, and implied that increased DEPTOR impaired the commitment of chondrocytes to hypertrophy (Csukasi et al., 2018). DEPTOR has also been suggested as an inhibitor of osteogenesis (Chen et al., 2018) and a promoter of adipogenesis in white adipose tissue (WAT) (Laplante et al., 2012). We therefore studied DEPTOR expression in control and JMC and BOCD bones. IHC showed that DEPTOR was highly expressed in the BM and trabeculae of control bone (Figure 1M); in JMC bone, DEPTOR expression was observed in the adipocytes populating the BM, in the ectopic regions of cartilage located in the diaphysis as well as late hypertrophic chondrocytes (Figures 1N, P). In contrast, BOCD patients showed expression of DEPTOR in the BM and trabecular bone but no expression was detected in the ectopic chondrocytes invading the trabecula (Figures 1O, Q).

**FIGURE 2:**
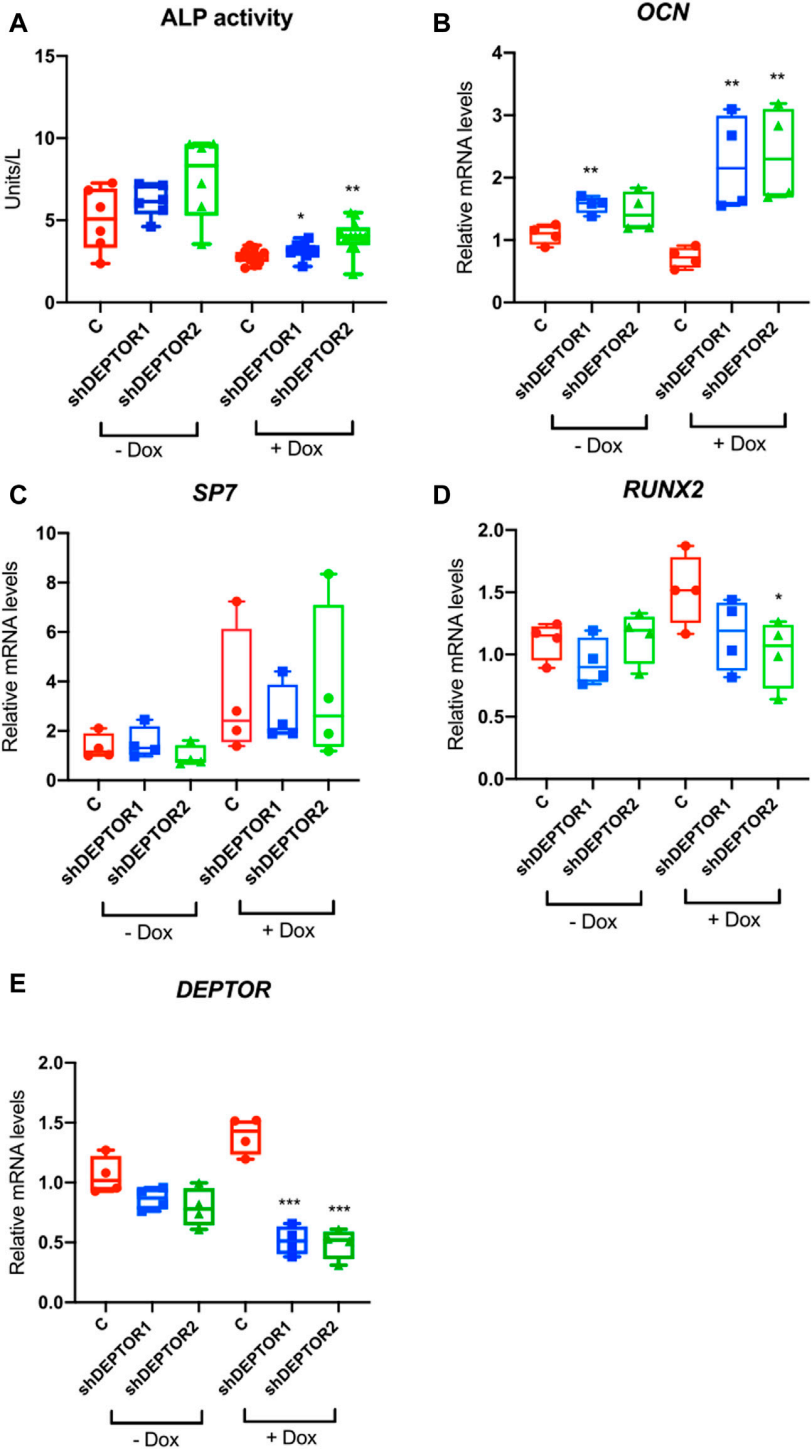
Knock down of DEPTOR promotes osteogenic differentiation of MSC. **(A)** ALP activity of Control **(C)** and shRNA DEPTOR lines (shDEPTOR1 and shDEPTOR2) with (right) and without (left) dox. Without dox *N* = 6, with dox *N* = 12. Cells were grown using osteogenic induction media for 7 days after which ALP was measured. **(B–D)** Gene expression analysis of osteogenesis marker genes in Control and shDEPTOR1/2 lines with and without dox: *OCN* **(B)**, *SP7* **(C)**, *RUNX2* **(D)** and *DEPTOR* **(E)** as measured by qPCR. Cells were left to differentiate using osteogenic medium for 7 days. Graphs represent means ± SEM. *N* = 4. Student’s *t*-test, *p < .05, ** *p* < .01. Dox: doxycycline; *OCN*: osteocalcin; *SP7*: Osterix.

In order to directly study the role of DEPTOR in skeletal progenitor differentiation, we performed a stable knockdown of DEPTOR in an immortalized mesenchymal stem cell (MSC) human cell line, using a doxycycline (Dox) inducible shRNA system (shDEPTOR). To evaluate the effect of DEPTOR in osteogenic differentiation, we measured ALP activity in control and shDEPTOR lines after osteogenic differentiation. Knockdown of DEPTOR resulted in increased activity of ALP compared to control (Figure 2A). This was consistent with increased levels of expression of osteocalcin (*OCN*) (Figure 2B). No change was observed in the levels of expression of Osterix (*SP7*) (Figure 2C) whereas *RUNX2* expression was decreased, although significance was achieved only in one of the two lines (Figure 2D). These results imply a negative role of DEPTOR in osteogenic differentiation and also suggest that the effects of DEPTOR in osteogenesis may be stage-specific because differing results were observed in markers of early osteoblasts (*RUNX2*) and late osteoblasts (*OCN*). Another work linking DEPTOR with osteogenesis demonstrated that knock down of *DEPTOR* in primary MSC resulted in increased expression not only of *OCN* but also in *SP7* and *RUNX2* (Chen et al., 2018); this difference could be explained by the different cell lines used and maybe to the degree of *DEPTOR* knock down. It is important to note that treatment with Dox resulted in changes in ALP activity and also in the expression of some osteogenic genes (*OCN* and *RUNX2*). Dox is believed to induce some metabolic changes in cells that can affect proliferation and differentiation, which could explain its effect in ALP activity and gene expression. Despite these changes, because all samples were treated the same way, we consider that a comparison between Control and shDEPTOR1/2 Dox treated and Control and shDEPTOR1/2 untreated cells is reliable, but caution is needed in the interpretation of results.

### DEPTOR controls TAZ subcellular localization and degradation

**FIGURE 3:**
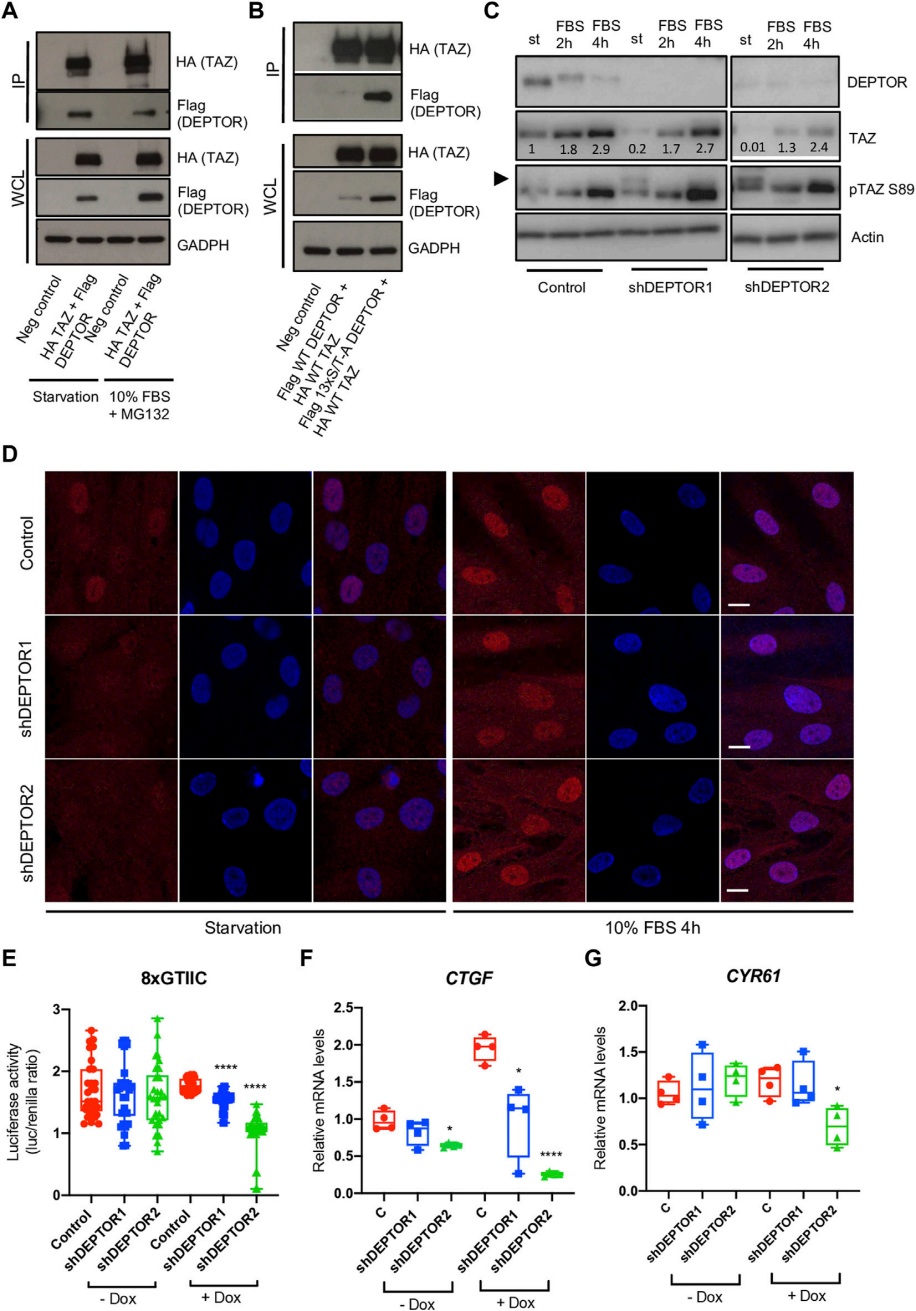
DEPTOR controls TAZ subcellular localization. **(A–B)** Co-IP of DEPTOR and TAZ. **(A)** N-terminal HA tagged TAZ and N-terminal Flag tagged DEPTOR were cotransfected into HEK293T cells. After transfection cells were starved O/N and then treated with 10% FBS and MG132 for 4 hours. TAZ was pulled down using anti-HA magnetic beads and the interaction with DEPTOR was detected by western blot using an anti-Flag antibody. **(B)** N-terminal HA tagged TAZ and N-terminal Flag tagged 13xS/T-A DEPTOR were cotransfected into HEK293T cells. After transfection cells were starved O/N. IP was performed as in **(A)**. **(C)** Western blots of Control and shDEPTOR1/2 lines treated with Dox. Cells were starved O/N and then treated with 10% FBS for two and 4 hours. Numbers indicate quantification of bands using Fiji. **(D)** Immunofluorescence of TAZ in Dox treated Control and shDEPTOR1/2 lines under starvation and after 4 h 10% FBS treatment. **(E)** Luciferase reporter assay of YAP/TAZ reporter (8xGTIIC) in Control and shDEPTOR1/2 lines under starvation. Graphs represent means ± SEM. *N* = 30. Student’s *t*-test, ****p < .0001. **(F–G)** Gene expression analysis of YAP/TAZ target genes CTGF **(F)** and CYR61 **(G)** under starvation, with and without dox as measured by qPCR. Graphs represent means ± SEM. *N* = 4. Student’s *t*-test, *p < .05, ****p < .0001. Dox, doxycycline. Scale bars: 15 μm.

The member of the Hippo pathway, TAZ, is a known regulator of MSCs differentiation (Heng et al., 2020). TAZ was initially reported to directly activate the transcriptional activity of RUNX2, inducing osteogenesis, while inhibiting the transcriptional activity of PPARγ, therefore repressing adipogenesis (Hong et al., 2005). But the role of TAZ and YAP (another member of the Hippo pathway that has overlapping roles with TAZ) in bone development have been controversial, depending on the stage when YAP and TAZ are deleted, both promotion or inhibition of bone formation have been observed (Kegelman et al., 2018; Xiong et al., 2018; Kegelman et al., 2020). TAZ is a PDZ binding protein (Hong et al., 2005) and DEPTOR is a DEP-domain protein that contains a PDZ domain (Chen and Hamm, 2006; Consonni et al., 2014; Caron et al., 2018), therefore we speculated that DEPTOR might interact with TAZ. DEPTOR is phosphorylated and subsequently degraded by the proteasome in the presence of serum (Peterson et al., 2009). We also showed that the increased levels of DEPTOR found in JMC cells were only found under serum starvation and after short exposures to serum (Csukasi et al., 2018). Therefore, we followed the same strategy, we serum starved the cells O/N and then treated them with 10% serum for 4 hours together with the proteasome inhibitor MG132 to analyze the response to serum but at the same time to avoid the serum-induced degradation of DEPTOR. We tested the hypothesis that there was an interaction between DEPTOR and TAZ by using co-immunoprecipitation (Co-IP). TAZ and DEPTOR clearly interacted both under conditions of starvation and serum treatment. The interaction was stronger under starvation conditions, even when DEPTOR and TAZ total levels were higher under serum treatment with the proteasome inhibitor (Figure 3A, Supplementary Figure S1A). To study the dynamics of the interaction in more detail we used a non-phosphorylatable version of DEPTOR (13xS/T-A). DEPTOR 13xS/T-A cannot be phosphorylated and therefore is not degraded by the proteasome, leading to cellular accumulation. Our results showed that DEPTOR interacted with TAZ in its un-phosphorylated state (Figure 3B, Supplementary Figure S1B). The interaction between the un-phosphorylated DEPTOR with TAZ was consistent with the WT DEPTOR detected interaction under conditions of serum starvation.

TAZ is a transcriptional regulator that shuttles between the nucleus and cytoplasm depending on its phosphorylation state (Kofler et al., 2018). To understand how DEPTOR interaction affected TAZ function we analyzed the levels of TAZ in the shDEPTOR lines under serum starvation and after two and 4 h of 10% serum treatment respectively. We first confirmed that serum treatment induced the phosphorylation and degradation of DEPTOR as observed by a shift in the migration of DEPTOR in the gel and a decrease in signal (Figure 3C). We also detected the increased expression of TAZ after serum stimulation that has previously been described (Yu et al., 2012b). Knockdown of DEPTOR resulted in decreased levels of total TAZ and increased levels of its phosphorylated form (pSer89, measured using a specific antibody for the phosphorylated form) under starvation conditions, but not with serum stimulation (Figure 3C, Supplementary Figures S1C–S1D). The phosphorylation of Ser89 in TAZ is correlated with its interaction with 14-3-3 proteins and therefore its retention in the cytoplasm, thus increased phosphorylation in this residue suggests increased retention in the cytoplasm. These results suggest that DEPTOR may be involved in TAZ degradation and subcellular localization. To confirm that DEPTOR regulates TAZ subcellular localization, we performed an immunofluorescence of TAZ in control and shDEPTOR lines. We observed more cytoplasmic localization of TAZ in the shDEPTOR lines compared to the control under starvation, while serum treatment restored the normal subcellular localization of TAZ (Figure 3D, Supplementary Figures S1E–S1G). We next measured the transcriptional activity of TAZ under starvation in control and shDEPTOR lines using a luciferase reporter (8xGTIIC); it is important to note that this reporter measures the activity of both TAZ and YAP. The YAP/TAZ luciferase activity was significantly decreased in the shDEPTOR lines compared to control (Figure 3E). Moreover, the expression of the YAP/TAZ target genes *CTGF* and *CYR61* was also decreased, although *CYR61* only reached significance in the shDEPTOR2 line (Figures 3F, G). These results are consistent with an increased retention of TAZ in the cytoplasm in the absence of DEPTOR. To determine if DEPTOR also regulated YAP we measured YAP levels in the DEPTOR KD lines and we observed no change in YAP protein accumulation in the shDEPTOR lines compared to control (Supplementary Figure S2A). We also analyzed YAP subcellular localization and again found no correlation between YAP localization and DEPTOR levels (Supplementary Figure S2B). These results suggest that DEPTOR specifically regulates TAZ and has no effect on YAP.

### DEPTOR regulates TAZ through its interaction with DVL2

**FIGURE 4:**
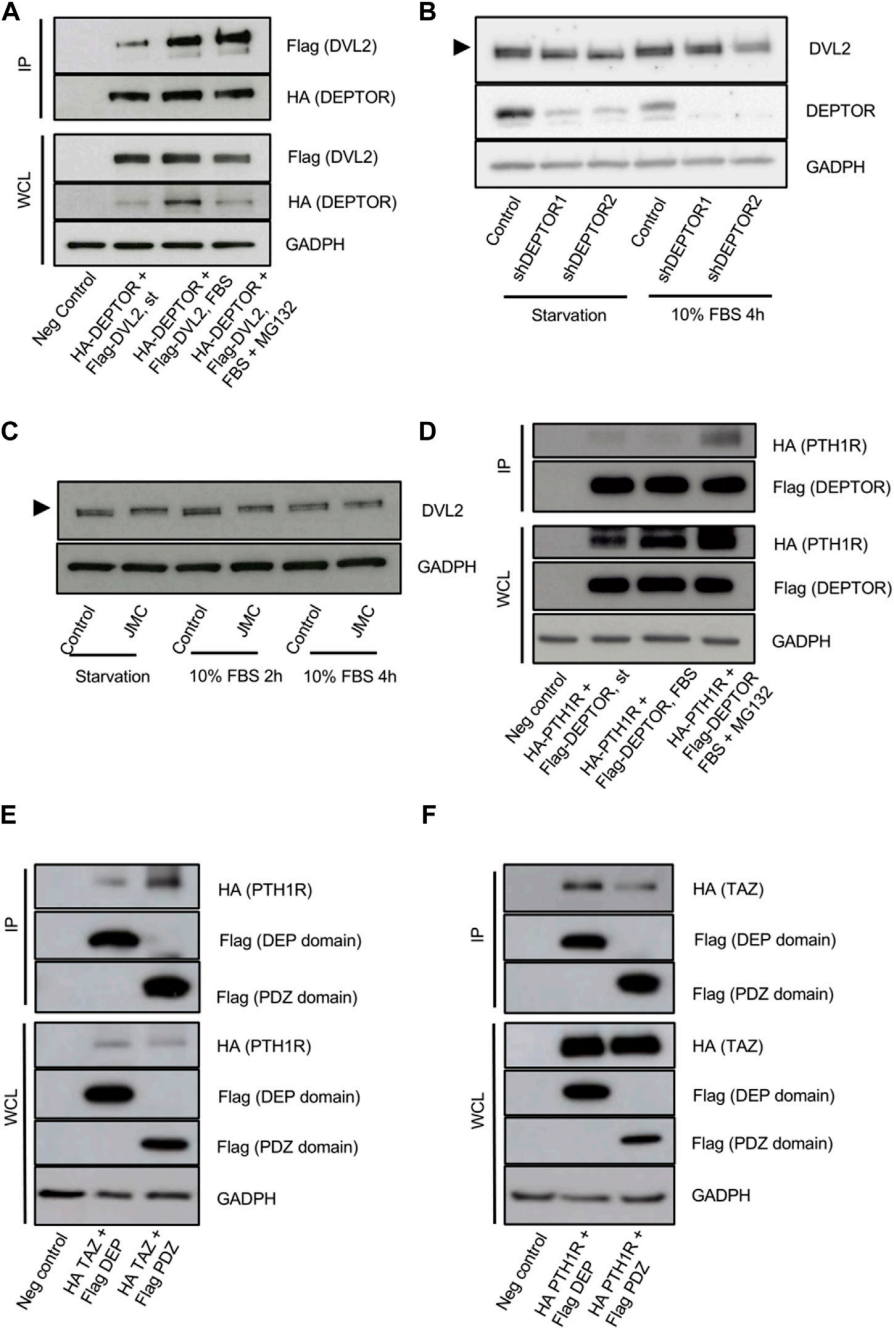
DEPTOR-DVL2 interaction regulates TAZ shuttling between nucleus and cytoplasm. **(A)** Co-IP of DEPTOR and DVL2. N-terminal HA tagged DEPTOR and N-terminal Flag tagged DVL2 were cotransfected into HEK293T cells. After transfection cells were starved O/N and then treated with 10% FBS alone or in combination with MG132 for 4 h. DEPTOR was pulled down using anti-HA magnetic beads and the interaction with DVL2 was detected by western blot using an anti-Flag antibody. **(B)** Western blots of Control and shDEPTOR1/2 lines. Cells were starved O/N and then treated with 10% FBS for 4 h. **(C)** Western blot of DVL2 in Control and JMC fibroblasts. Cells were starved O/N and then treated with 10% FBS for two and 4 hours. **(D)** Co-IP of DEPTOR and PTH1R. N-terminal HA tagged PTH1R and N-terminal Flag tagged DEPTOR were cotransfected into HEK293T cells. After transfection cells were starved O/N and then treated with 10% FBS alone or in combination with MG132 for 4 h. DEPTOR was pulled down using anti-Flag magnetic beads and the interaction with PTH1R was detected by western blot using an anti-HA antibody. **(E–F)** Co-IP of PTH1R or TAZ and DEPTOR DEP and PDZ domains. **(E)** N-terminal HA tagged PTH1R and N-terminal Flag tagged DEP or PDZ domains were cotransfected into HEK293T cells. After transfection cells were starved O/N and then treated with 10% FBS for 4 h. DEP and PDZ domains were pulled down using anti-Flag magnetic beads and the interaction with PTH1R was detected by western blot using an anti-HA antibody. **(F)** N-terminal HA tagged TAZ and N-terminal Flag tagged DEP or PDZ domains were cotransfected into HEK293T cells. After transfection cells were starved O/N. DEP and PDZ domains were pulled down using anti-Flag magnetic beads and the interaction with TAZ was detected by western blot using an anti-HA antibody.

The increased cytoplasmic localization of TAZ in the DEPTOR KD lines was unexpected because it suggests that DEPTOR promotes TAZ nuclear localization. Due to DEPTOR’s protein characteristics and its known localization in the cytoplasm, we expected DEPTOR to act by retaining TAZ in the cytoplasm. Because of this we speculated that DEPTOR’s control over TAZ subcellular localization might also be indirect, through the control of another protein that regulates TAZ localization in the cytoplasm. One candidate was Dishevelled (DVL), a protein involved in canonical and non-canonical WNT signaling. DVL has been shown to interact with phosphorylated TAZ, preventing its translocation to the nucleus (Varelas et al., 2010). Because DVL is a DEP-domain protein, similar to DEPTOR (Chen and Hamm, 2006; Consonni et al., 2014), we hypothesized that DEPTOR also interacted with DVL to indirectly control TAZ subcellular localization. To test this hypothesis, we first tested whether DEPTOR and DVL physically interacted. DEPTOR and DVL2 were co-expressed in HEK293T cells. Cells were serum starved O/N and then treated with 10% serum alone for 4 h or with 10% serum together with the proteasome inhibitor MG132. We found a clear interaction between DEPTOR and DVL2 in the three conditions analyzed although the interaction was stronger in the presence of serum compared to starvation (Figure 4A, Supplementary Figure S2A). We also observed a shift in DVL2 migration due to its interaction with DEPTOR (Figure 4A), suggesting that the interaction of DVL2 with DEPTOR promotes its phosphorylation. We next measured DVL2 in the shDEPTOR lines; we observed decreased levels of phosphorylation of DVL2 in the shDEPTOR lines compared to control evidenced by decreased intensity of the upper band (arrow) only under starvation, while serum treatment restored the phosphorylation of DVL2 levels to normal (Figure 4B, Supplementary Figure S3B). We also measured DVL2 in JMC cells, where DEPTOR levels are high, and observed accumulation of the phosphorylated form of DVL2 in patient cells compared to control (upper band) again only under conditions of starvation (Figure 4C). These results confirm that DEPTOR regulates DVL2 phosphorylation through a direct interaction; changes in DVL2 phosphorylation determine whether TAZ localizes to the nucleus or the cytoplasm.

To fully dissect the molecular mechanism that links DEPTOR with the changes observed in skeletal differentiation of patients with mutations in PTH1R, we investigated whether DEPTOR also directly interacted with PTH1R. We co-expressed PTH1R and DEPTOR in HEK293T cells, starved cells O/N and then treated them with serum alone or in combination with MG132 for 4 h. We were able to detect an interaction between DEPTOR and PTH1R that was stronger in the presence of serum (in combination with MG132) (Figure 4D, Supplementary Figure S2B). This result suggests that DEPTOR is involved in transmitting the PTH/PTHrP signals.

A deeper analysis of the interaction between DEPTOR and TAZ and DEPTOR and PTH1R demonstrated that DEPTOR interaction with PTH1R was mostly done through its PDZ domain (Figure 4E, Supplementary Figure S2C); on the contrary DEPTOR interacted with TAZ more strongly through its DEP domain (Figure 4F, Supplementary Figure S2D).

Altogether, our results establish DEPTOR as a critical regulator of TAZ subcellular localization and therefore its transcriptional activity and demonstrate that DEPTOR regulation of TAZ is achieved through the control of DVL2.

## Discussion

In this study, we describe aberrant differentiation of skeletal progenitors leading to accumulation of fat or appearance of ectopic cartilage in two different but related skeletal genetic disorders, JMC and BOCD. These diseases are both caused by mutations in the PTH/PTHrP receptor, PTH1R, but JMC is an autosomal dominant disorder that results from constitutive activation of the PTH1R receptor (Schipani et al., 1995), whereas BOCD is an autosomal recessive disease that leads to loss-of-function of PTH1R (Jobert et al., 1998). JMC shows characteristics of delayed chondrocyte and bone formation, on the contrary BOCD has been described as a disease with advanced bone maturation (Jobert et al., 1998; Kozlowski et al., 1999). But to our knowledge, no in-depth analysis of the bone phenotype observed in these two disorders has been performed. We demonstrate that JMC bones are filled with fat and show a decrease in trabecular bone. Interestingly, patient bones also show ectopic cartilage islands within the bone marrow-trabecular diaphyseal space, completely surrounded by fat. This phenotype is consistent with a dysregulation of skeletal progenitors differentiation because progenitors can differentiate into bone, cartilage and fat, a process that is tightly regulated by genetic and environmental factors. Skeletal stem cells dysregulation leads to aberrant bone/fat balance, characteristic of several diseases, both genetic (progressive osseous hyperplasia and fibrodysplasia ossificans progressiva) and chronic (aging, immobility, anorexia nervosa and osteoporosis (Devlin and Rosen, 2015; Veldhuis-Vlug and Rosen, 2017)). The coexistence of differentiation to fat and cartilage in these patients indicates that the genetic effects are also environmentally regulated, something in the specific regions of the bone must be switching differentiation towards one tissue or the other. It also highlights the complexity of the process of progenitors differentiation and the fact that much more work is needed to fully understand this process. Loss-of-function mutations of PTH1R found in BOCD result in a loss of boundary between bone and cartilage compartments around the growth plate region. Based on our results, we could conclude that hyperactivation of PTH1R signaling favors differentiation to fat, whereas inhibition of this pathway switches differentiation towards the production of cartilage. But the presence of a region of chondrocytes immersed in the fat of JMC bones, shows that the effects of these mutations are more complex and the cell fate of each progenitor cell is likely complex. It is also interesting to note that PTHrP haploinsufficient mice and conditional deletion of PTH1R in mouse skeletal progenitors, using Prx-Cre, resulted in accumulation of adipocytes in bones (Amizuka et al., 1996; Fan et al., 2017), a similar histologic phenotype observed with the constitutive activation of PTH1R in JMC. There was no mention of a differentiation towards cartilage in none of these models. This could be due to the anatomical differences between mouse and human in the diaphyseal space, which accumulates a more intricate trabecular ossification. Differences in the phenotypes found between mouse and human highlight the importance of studying multiple model systems to fully understand how biological processes are controlled in different species. Yet, it is clear that both mouse and humans with dysregulated PTH/PTHrP signaling show aberrant differentiation of skeletal progenitors which leads to unbalanced formation of skeletal tissues.

We had previously demonstrated increased levels of DEPTOR, a mTOR inhibitor, in JMC cells and that misexpression of DEPTOR in the growth plate correlated with compromised commitment of chondrocytes to hypertrophy (Csukasi et al., 2018). Unfortunately, we were unable to measure the levels of DEPTOR in BOCD because cells from these patients were unavailable, however IHC showed low expression of DEPTOR in BOCD, which was restricted to bone, chondrocytes showed almost complete absence of DEPTOR, contrary to what is observed in JMC where DEPTOR showed a clear accumulation in growth plate chondrocytes (Csukasi et al., 2018). To directly correlate DEPTOR to skeletal cells differentiation, we knocked down DEPTOR in a human MSC line, which resulted in increased ALP activity and increased expression of the bone marker *OCN*, suggesting that DEPTOR acts as an inhibitor of osteogenesis, and again consistent with the patient phenotypes. Increased ALP activity in DEPTOR knock down in BMSC has been previously described (Chen et al., 2018), this study suggested that DEPTOR acts as a negative regulator of osteogenesis and linked high DEPTOR levels to the progress of osteoporosis, where fat accumulation has been documented (Devlin and Rosen, 2015; Veldhuis-Vlug and Rosen, 2017). Although DEPTOR control over the adipogenic lineage commitment of BMSC has not been assessed previously, overexpression of DEPTOR in mice promotes white adipose tissue (WAT) accumulation (Laplante et al., 2012). Moreover, the levels of DEPTOR increase during adipogenic differentiation of mouse embryonic fibroblasts (MEFs) and 3T3-L1 cells, indicating that DEPTOR has a role in promoting adipogenesis (Laplante et al., 2012). Our results now indicate that DEPTOR also promotes adipogenesis of BMSC, when increased due to Mendelian disorders.

Our work provides a deep understanding of the molecular mechanism by which DEPTOR regulates skeletal progenitor differentiation. DEPTOR was identified in 2009 as a component of mTORC1/2 complexes that acts as a negative regulator of mTOR activity (Peterson et al., 2009). However, evidence is emerging that suggest that DEPTOR has roles that are independent of mTOR (Catena et al., 2016; Li et al., 2021). We show that DEPTOR interacts with and effects localization of the transcriptional regulator TAZ, implicating DEPTOR in an unappreciated signaling pathway. DEPTOR regulates the shuttling of TAZ between the cytoplasm and the nucleus, therefore modulating its transcriptional activity. DEPTOR acts in concert with DVL2 to further retain TAZ in the cytoplasm under nutrient deprivation, preventing its transcriptional control over its targets. Under nutrient sufficiency, DEPTOR-DVL2 release TAZ, which translocates to the nucleus to regulate transcription. When DEPTOR releases TAZ, it directly interacts with PTH1R to regulate PTH and WNT signaling (Supplementary Figure S3). Several evidence supports this scenario: -DEPTOR directly interacts with DVL2 and TAZ; -DEPTOR interaction with DVL2 affects its phosphorylation; -DEPTOR KD cells show decreased phosphorylation of DVL2 whereas JMC cells show the opposite; -DEPTOR KD cells show expression of TAZ mostly in the cytoplasm and increased phosphorylation of TAZ (S89) consistent with its retention in the cytoplasm; -DEPTOR KD cells show decreased activity of a YAP/TAZ transcriptional reporter and decreased expression of the YAP/TAZ target genes *CTGF* and *CYR61*; -DEPTOR directly interacts with PTH1R.

TAZ was identified as a regulator of MSC differentiation in 2005, when a group showed that TAZ directly activated RUNX2 while inhibiting PPARγ transcriptional activities; morpholino deletion of TAZ in zebrafish decreased bone formation (Hong et al., 2005). Because of these results TAZ was proposed as a promoter of osteogenesis and an inhibitor of adipogenesis. Since then, conflicting results have been published by different groups regarding TAZ regulation of osteogenesis. TAZ is a member of the Hippo pathway and acts in concert with YAP, their activities are partially redundant. TAZ KO mice show only minor skeletal abnormalities, a slightly shorter skeleton was described in the KO compared to WT (Hossain et al., 2007). Prx-Cre mediated deletion of both YAP and TAZ resulted in embryonic lethality; deletion of both copies of TAZ and only one of YAP using Prx-Cre resulted in increased bone formation (Xiong et al., 2018). On the contrary, deletion of YAP and TAZ from mature osteoblasts and osteocytes using Osterix-Cre and Dmp1-Cre reduced bone formation (Kegelman et al., 2018; Xiong et al., 2018; Kegelman et al., 2020). These results suggest that in skeletal progenitors, YAP and TAZ oppose differentiation towards the osteoblast lineage whereas in mature osteoblasts and osteocytes they promote bone formation. This is consistent with our results; DEPTOR KD MSC cells show retention of TAZ in the cytoplasm under osteogenic differentiation, leading to increased ALP activity and expression of *OCN*, indicating that in skeletal progenitors, TAZ inhibits osteogenic differentiation.

In conclusion, we describe in detail the molecular mechanism by which DEPTOR controls skeletal progenitors differentiation towards bone or fat through the analysis of two genetic skeletal disorders with mutations in PTH1R. The regulation of TAZ by DEPTOR points to a role of DEPTOR completely independent of mTOR and identifies a new pathway regulated by this molecule. The identification of DEPTOR as a key regulator of the pathologic accumulation of fat in bones could have major implications in the treatment of skeletal diseases that show increased fat/bone ratio such as aging osteoporosis.

While in the process of publishing our findings, Ouyang, et al. demonstrated that knockout of DEPTOR in mice using Prx-Cre resulted in decreased accumulation of fat in bone after ovariectomy (Ouyang et al., 2022). They also showed that DEPTOR interaction with TAZ was responsible for the phenotype observed (Ouyang et al., 2022). Our work complements these previous findings showing that dysregulation of DEPTOR levels in humans due to two different mutations in the PTH/PTHrP receptor, PTH1R, also result in aberrant differentiation of skeletal progenitors leading to accumulation of fat or cartilage in the bones of patients with JMC and BOCD. We also describe a mechanism of disease based on DEPTOR interaction with TAZ but we provide evidence that DEPTOR regulation of TAZ involves DVL2, a member of canonical and non-canonical WNT signaling, and demonstrate that DEPTOR directly interacts with PTH1R, linking DEPTOR effects to WNT and PTH/PTHrP signaling. Together, Ouyang, et al. and our own work provide clear evidence for a role of DEPTOR in skeletal progenitors differentiation.

## Materials and methods

### Ethics and patient information

This study was approved by the University of California at Los Angeles Institutional Review Board under protocol number IRB#14-00017. was obtained from all participants. Patients and their unaffected family members were ascertained under the above IRB-approved human subject’s protocol. Clinical information and imaging were obtained from review of all available medical records. BOCD and JMC were given a firm diagnose of the disease based on clinical, radiographic and histological findings. BOCD patient: neonatal death; JMC: died at 3 years old.

### Cell culture

Dermal fibroblast cultures were established from explanted skin biopsies from the individual affected with JMC (International Skeletal Dysplasia Registry reference number R93-393) and controls. All cells were grown in Dulbecco-Vogt–modified Eagle medium (DMEM) supplemented with 10% FBS.

The immortal MSC cell line used was ASC52telo (catalog no. SRC-4000, ATCC). Cells were grown in MSC basal medium (catalog no. PCS-500-030, ATCC), supplemented with 5 ng/mL FGF basic, 5 ng/mL FGF acidic, 5 ng/mL EGF, 2% FBS, 2.4 mM glutamine.

For serum treatments, cells were starved overnight (no serum) and then treated with the same media supplemented with 10% FBS for the indicated times.

For protein analyses, cells were collected in IP lysis buffer (catalog no. 87787, Thermo Fisher Scientific) supplemented with proteinase inhibitors and concentrations were determined using the Pierce™ BCA Protein Assay Kit (catalog no. 23227, Thermofisher).

Osteogenic induction was performed using DMEM, supplemented with 10% FBS, 100 μM ascorbic acid, 2 mM β-glycerophosphate and 10 nM dexamethasone for 7 days, medium was changed every other day.

### Western blot and IPs

For Western blot analyses, protein lysates were separated by electrophoresis on 10% or gradient (4%–20%) SDS–polyacrylamide gels, transferred to polyvinylidene difluoride membranes, blocked in 5% milk, and probed with primary antibodies: anti-DEPTOR antibody (1:1,000; catalog no. 11816, CST), anti-TAZ antibody (1:1,000; catalog no. 70148, CST), anti-pTAZ S89 (1:1,000; catalog no. 59971, CST), anti-DVL2 (1:1,000; catalog no. 3224, CST), anti-GAPDH (1:2000; catalog no. 2118), anti-YAP (1:1,000); catalog no. 14074, CST), anti-Actin (1:2,000; catalog no. 3700), anti-HA (1:1,000; catalog no. 3724), anti-Flag (1:1,000; catalog no. 14793). Peroxidase-conjugated secondary antibodies (catalog nos. 7071 and 7072, CST) were used, and immunocomplexes were identified using the ECL (enhanced chemiluminescence) Detection Reagent (catalog no. 322009, Thermofisher) or Super Signal West Femto (catalog no. 34094, Thermofisher). Fiji was used to quantify bands after gel analysis recommendations from ImageJ and (http://rsb.info.nih.gov/ij/docs/menus/analyze.html#gels). Experiments were replicated at least three times.

For IPs, HEK293T cells were transfected with the appropriate vetors: HA-DEPTOR (catalog no. 73390, Addgene), Flag-DEPTOR (catalog no. 34610, Addgene), Flag-DEPTOR 13xS/T-A (catalog no. 21702, Addgene), Flag-DEPTOR DEP domain (catalog no. 21700, Addgene), Flag-DEPTOR PDZ domain (catalog no. 21701, Addgene), HA-TAZ (catalog no. 32839, Addgene). HA-PTH1R was synthesized by Genescript. 48 h after transfections, cells were harvested and protein extracts were immunopurified using anti-FLAG magnetic beads (catalog no. M8823, Merck) or anti-HA magnetic beads (catalog no. 88837, Thermofisher).

### Transfections and viral infections

Cells were transfected using Lipofectamine™ 3000 Transfection Reagent (catalog no. L3000015, Thermofisher), according to manufacturer’s instructions. Proteins were harvested after 48 h.

8xGTIIC (catalog no. 34615, Addgene) lentiviral particles were produced at the Vector Core Facility at UCLA Center for Systems Biomedicine (https://www.uclahealth.org/gastro/csbm/vector-core). Briefly, lentivirus-based vectors were generated by transient cotransfection of HEK293T cells with a three-plasmid combination, as described previously, with slight modifications (Naldini et al., 1996). The construct pMD.G was used for the production of the VSV-G viral envelope in combination with the packaging constructs PAX2 (Catalog no. 12260, Addgene) and pRSV–REV. Briefly, 10 cm dish of non-confluent HEK293T cells were co-transfected with PAX2, pMDG (encoding the VSV-G envelope), pRSV–REV and the pRRL-XYZ (8xGTIIC), by the CaPi-DNA coprecipitation method (Chen and Okayama, 1987). Next day, the medium was adjusted to make a final concentration of 10 mM sodium butyrate and the cells were incubated for 8 h to obtain high-titer virus production as previously described (Sakoda et al., 1999). After the 8 h incubation, cells were washed and incubated in fresh medium without sodium butyrate. Conditioned medium was harvested 16 h later and passed through .45 mm filters. Viral titer was determined by assessing viral p24 antigen concentration by ELISA (the Alliance^®^ HIV-I p24 ELISA Kit, Perkin Elmer) and hereafter expressed as μg of p24 equivalent units per milliliter.

### Generation of DEPTOR shRNA lines

For shRNA, the ASC52telo cells with stably integrated, doxycycline-inducible expression of shRNA targeting DEPTOR mRNA coding sequence (CDS, shDEPTOR1) or 3′UTR (shDEPTOR2), and the respective scramble controls were generated by lentiviruses as previously described (Bosakova et al., 2019; Bosakova et al., 2020). Lentiviral vector containing doxycycline-inducible U6 promoter and TetRep-P2A-Puro-P2A-mCherry (Peskova et al., 2019) (kindly provided by Mikael Altun) was modified to express shRNA by introducing the following oligonucleotides (DEPTOR-CDS forward: CCG​GTC​AAA​CTC​TTC​TAC​CGC​TTT​ACT​CGA​GTA​AAG​CGG​TAG​AAG​AGT​TTG​ATT​TTT​G, DEPTOR-CDS reverse: AAT​TCA​AAA​ATC​AAA​CTC​TTC​TAC​CGC​TTT​ACT​CGA​GTA​AAG​CGG​TAG​AAG​AGT​TTG​A; DEPTOR-3′UTR forward: CCG​GCC​TAC​ATG​ATA​GAA​CTG​CCT​TCT​CGA​GAA​GGC​AGT​TCT​ATC​ATG​TAG​GTT​TTT​TG, DEPTOR-3′UTR reverse: AAT​TCA​AAA​AAC​CTA​CAT​GAT​AGA​ACT​GCC​TTC​TCG​AGA​AGG​CAG​TTC​TAT​CAT​GTA​GG; scrambled forward: CCG​GAT​CTC​TCT​TAT​ACT​ACG​CCA​TCT​CGA​GAT​GGC​GTA​GTA​TAA​GAG​AGA​TTT​TTT​G scrambled reverse: AAT​TCA​AAA​AAT​CTC​TCT​TAT​ACT​ACG​CCA​TCT​CGA​GAT​GGC​GTA​GTA​TAA​GAG​AGA​T. Cloned shRNA sequences were verified by Sanger sequencing. Lentiviral particles were generated as described previously (Barta et al., 2016; Peskova et al., 2019) using pMD2.G (Catalog no. 12259, Addgene) and psPAX2 (Catalog no. 12260, Addgene) (gift from Didier Trono). After transduction, at least 2 × 104 mCherry-positive cells were sorted using BD FACSAria™ II (BD Biosciences). Generated cell lines were propagated in the presence of 1 μg/mL puromycin. shRNA expression was induced by 1 μg/mL doxycycline.

### Histological analyses and immunohistochemistry

For histology and immunocytochemistry, human (control, JMC and BOCD) tissues were fixed in 4% paraformaldehyde, decalcified using immunocal decalcification solution (catalog no. 1414-1, StatLab), and then paraffin-embedded. Paraffin blocks were sectioned at 10 μm. For histological analyses, sections were stained with Masson-Goldners.

For immunohistochemistry, paraffin sections were boiled for 20 min in Antigen Unmasking Solution (Vector Laboratories) and subsequently stained using the Rabbit-specific HRP/DAB (ABC) Detection IHC Kit (Abcam). Primary antibodies used were anti-DEPTOR (1:500; catalog no. 20985-1-AP, Proteintech), anti-AGGRECAN (catalog no. GTX54920, GeneTex) and anti-ALPL (catalog no. 4747 CST).

### Immunofluorescence and operetta

For immunofluorescence, cells were fixed in 4% paraformaldehyde for 10 min, permeabilized in .1% Triton for 10 min, followed by blockage in 10% goat serum for 1 h. Cells were incubated with anti-TAZ or anti-YAP primary antibodies (catalog no. 72804, CST; 1:100 and 14074, CST; 1:100) O/N at 4C, followed by secondary anti-rabbit Alexa Fluor 647 (catalog no. A32733TR, Invitrogen). Nucleus were stained using SlowFade Gold Antifade Mountant with DAPI (catalog no. S36942, Thermofisher). Samples were analyzed using a Leica SP5 confocal microscope equipped with a 40x HCX PL APO CS 1.30 NA UV oil immersion objective lens and high sensitivity HyD detectors. DAPI and AlexaFluor 647 were detected using 405nm and 633 nm lasers, with 408–556 nm and 638–753 nm spectral detection windows, respectively. Single optical sections were captured from the different samples using identical laser intensity and sensitivity settings. A non-confocal transmitted light channel was captured at the same time. Identical image processing and analysis steps were performed using FIJI/ImageJ.

For quantification of nuclear/cytoplasmic localization, an Operetta High-Content Screen system (Perkin Elmer) was used. Cells were seeded in a CellCarrier-96 plate (catalog no. 6005550, PerkinElmer) to 80% confluence with and without Dox. 48 h later, cells were serum-starved O/N and the above protocol of IF was performed. Plates were then scanned, and images were collected using the Operetta High Content Screening imaging system (Perkin Elmer) at 10 × magnification with 10 scattered fields of view. Images were analyzed with Harmony 4.8 software from Perkin Elmer. Nuclear areas were defined by DAPI staining, and TAZ fluorescence was measured in the defined region of interest (ROI). The cytoplasmic ROI fluorescence was measured by a dilation from the nuclear boundary.

### ALP activity

ALP activity was measured using SIGMAFAST™ p-Nitrophenyl phosphate Tablets (catalog no. N1891, Merck). Substrate was prepared according to manufacturer’s instructions and mixed with samples. Incubation was performed at 37C for 30 min. Absorbance was measured at 405 nm. Activity was calculated using the following formula

(ΔAbs/min) x Volume x 1,000

18.8 x light passage x volume

#### RNA extraction and qPCR

RNA was extracted using TRIzol™ Reagent (catalog no. 15596026, Thermofisher). Complementary DNA (cDNA) was prepared from 1 μg of RNA using PrimeScript™ RT Master Mix (catalog no. RR036A, Takara). qPCR was performed using TB Green Premix Ex Taq™ (catalog no. RR420L, Takara). Gene expression was calculated using the 2^−ΔΔCT^ method of analysis against the stable housekeeping gene TBP. Four biological replicates were performed with two technical replicates each. qPCR primers were: OCN, 5′- CTC​ACA​CTC​CTC​GCC​CTA​TT-3′ (forward), 5′- AAC​TCG​TCA​CAG​TCC​GGA​TT-3′ (reverse), RUNX2, 5′- GGC​AGT​TCC​CAA​GCA​TTT​CA-3′ (forward), 5′- AGG​TGT​GGT​AGT​GAG​TGG​TG-3′ (reverse), SP7, 5′- CAA​GGT​GTA​TGG​CAA​GGC​TT-3′ (forward), 5′- GCT​CAT​CCG​AAC​GAG​TGA​AC-3′ (reverse), CTGF, 5′- TAC​CAA​TGA​CAA​CGC​CTC​CT-3′ (forward), 5′- TGG​GAG​TAC​GGA​TGC​ACT​TT-3′ (reverse), CYR61, 5′- TCA​CCC​TTC​TCC​ACT​TGA​CC-3′ (forward), 5′- GTT​TTG​CTG​CAG​TCC​TCG​TT-3′ (reverse), DEPTOR, 5′-AGG​CAG​GTC​CAT​GAT​TCC​TC-3’ (forward), 5′-ATG​CTT​TTG​TTG​GTT​GGC​TG-3’ (reverse), TBP, 5′-CGG​CTG​TTT​AAC​TTC​GCT​TC-3’ (forward), 5′-CAC​ACG​CCA​AGA​AAC​AGT​GA-3’ (reverse).

#### Luciferase reporter assays

The 8xGTIIC viral particles were transduced into cells at 70% confluence. When cells reached confluence, they were starved O/N. Then, cells were lysed and luciferase activity was determined using a Dual-Luciferase Reporter Assay (catalog no. E1910, Promega).

#### Statistical analysis

GraphPad Prism was used for statistical analysis. All values are means ± SEM, as indicated in figure legends. All comparisons in the study were performed using Student’s *t*-test.

## Data Availability

The original contributions presented in the study are included in the article/Supplementary Material, further inquiries can be directed to the corresponding author.

## Supplementary material

SUPPLEMENTARY FIGURE S1
**(A–B)** Protein quantification of DEPTOR levels from Figure 3A**–**B. **(C–D)** Protein quantification of TAZ and pTAZ levels from Figure 3C. Proteins were quantified using Fiji. **(E–F)** The Operetta High-Content Screen system was used to calculate the ratios of fluorescence signals between the nucleus and cytoplasm in Control and shDEPTOR lines under starvation without **(E)** and with **(F)** Dox. Relative scored cells are presented as the average number of objects measured (*n* = 3 independent experiments). **(G)** Immunofluorescence of TAZ in Control and shDEPTOR1/2 lines without Dox, corresponding with Figure 3D. Cells were starved O/N and then treated with 10% FBS. Immunofluorescence was performed under starvation and after four hours 10% FBS treatment. Scale bars:10 μm.

SUPPLEMENTARY FIGURE S2
**(A)** Western blot of YAP, DEPTOR and GADPH in Control and shDEPTOR1/2 lines with and without Dox. Cells were starved O/N and then treated with 10% FBS for four hours. **(B)** Immunofluorescence of YAP in Control and shDEPTOR1/2 lines with and without Dox under O/N starvation conditions.

SUPPLEMENTARY FIGURE S3
**(A,C,D,E)** Protein quantifications, **(A)** DVL2, 702 **(C)** PTH1R, **(D)** PTH1R, **(E)** TAZ corresponding to western blots from Figure 4. Proteins were quantified using Fiji. **(B)** Western blot of DVL2, DEPTOR and GADPH in Control and shDEPTOR1/2 lines without Dox, corresponding with Figure 4B. Cells were starved O/N and then treated with 10% FBS for 4 h.

SUPPLEMENTARY FIGURE S4 Schematic representation of the mode of action of DEPTOR over TAZ, DVL2 and PTH1R. Under starvation, DEPTOR forms a complex with DVL2 that retains TAZ in the cytoplasm, preventing its transcriptional activity. In the presence of serum, DEPTOR-DVL2 release TAZ and interact with PTH1R; TAZ is translocated to the nucleus to control the expression of its target genes.
