# Supplementary material for: Skeletal diseases caused by mutations in PTH1R show aberrant differentiation of skeletal progenitors due to dysregulation of DEPTOR
Source: Front Cell Dev Biol. 2023 Jan 16;10:963389. doi: 10.3389/fcell.2022.963389 (PMC9885499; doi:10.3389/fcell.2022.963389)
Supplement: Supplementary file 1 [file DataSheet1.PDF]

S1

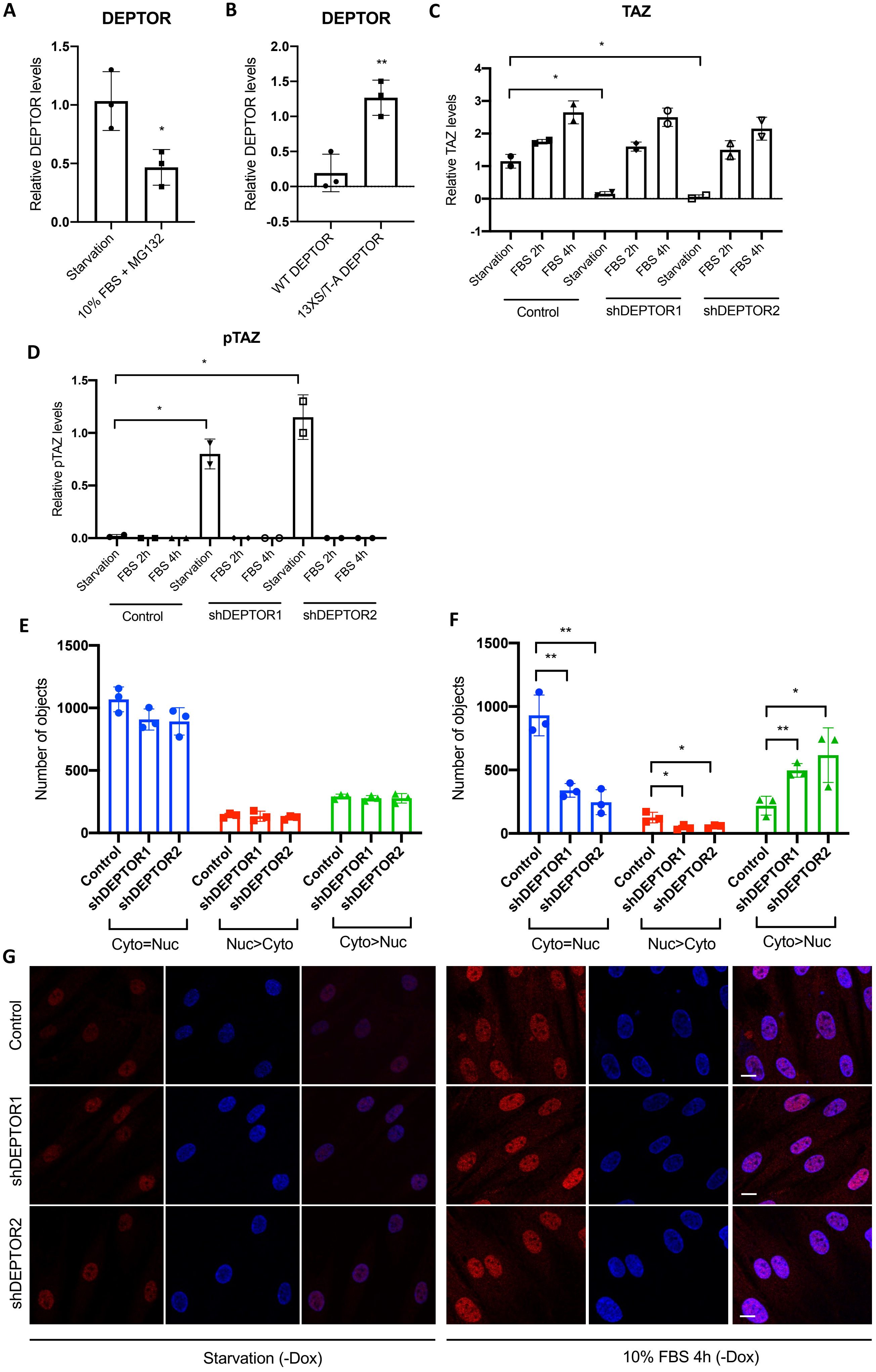
A
DEPTOR
Relative DEPTOR levels
1.5
1.0
0.5
0.0
*
Starvation
10% FBS + MG132
B
DEPTOR
Relative DEPTOR levels
2.0
1.5
1.0
0.5
0.0
-0.5
**
WT DEPTOR
13XS/T-A DEPTOR
C
TAZ
Relative TAZ levels
4
3
2
1
0
-1
*
*
Starvation
FBS 2h
FBS 4h
Starvation
FBS 2h
FBS 4h
Starvation
FBS 2h
FBS 4h
Control
shDEPTOR1
shDEPTOR2
D
pTAZ
Relative pTAZ levels
1.5
1.0
0.5
0.0
*
*
Starvation
FBS 2h
FBS 4h
Starvation
FBS 2h
FBS 4h
Starvation
FBS 2h
FBS 4h
Control
shDEPTOR1
shDEPTOR2
E
Number of objects
1500
1000
500
0
Control
shDEPTOR1
shDEPTOR2
Control
shDEPTOR1
shDEPTOR2
Control
shDEPTOR1
shDEPTOR2
Cyto=Nuc
Nuc>Cyto
Cyto>Nuc
F
Number of objects
1500
1000
500
0
**
**
*
*
*
**
Control
shDEPTOR1
shDEPTOR2
Control
shDEPTOR1
shDEPTOR2
Control
shDEPTOR1
shDEPTOR2
Cyto=Nuc
Nuc>Cyto
Cyto>Nuc
G
Control
shDEPTOR1
shDEPTOR2
Starvation (-Dox)
10% FBS 4h (-Dox)

S2

A

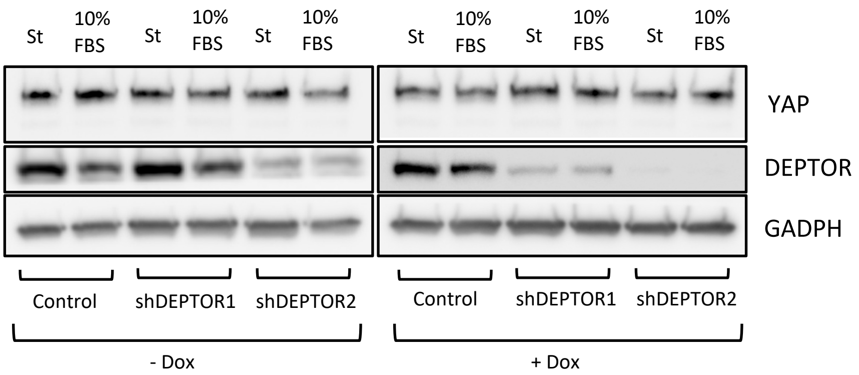

St
10% FBS
St
10% FBS
St
10% FBS
St
10% FBS
St
10% FBS
St
10% FBS
YAP
DEPTOR
GADPH
Control
shDEPTOR1
shDEPTOR2
Control
shDEPTOR1
shDEPTOR2
- Dox
+ Dox

B

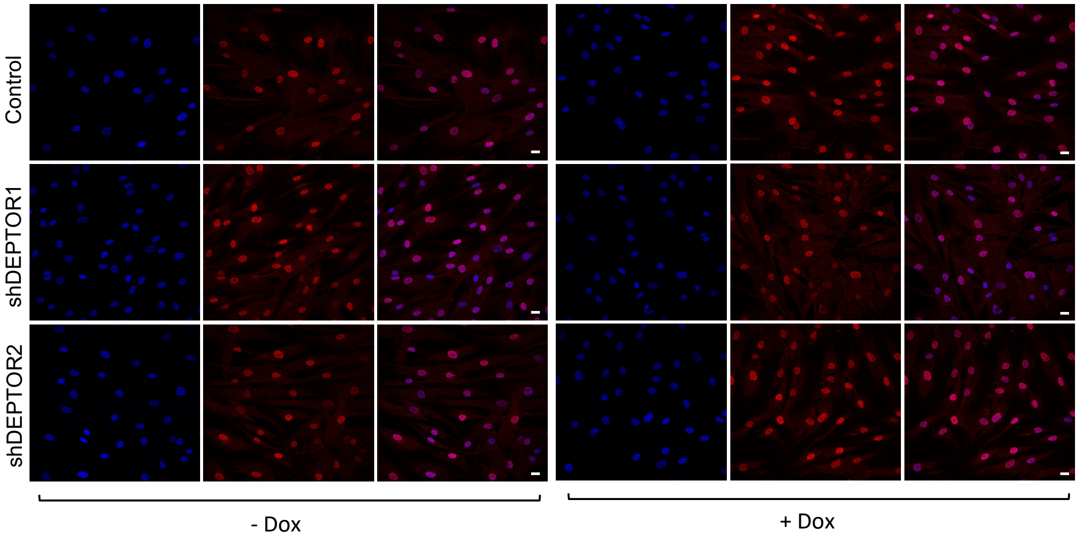

Control
shDEPTOR1
shDEPTOR2
- Dox
+ Dox

S3

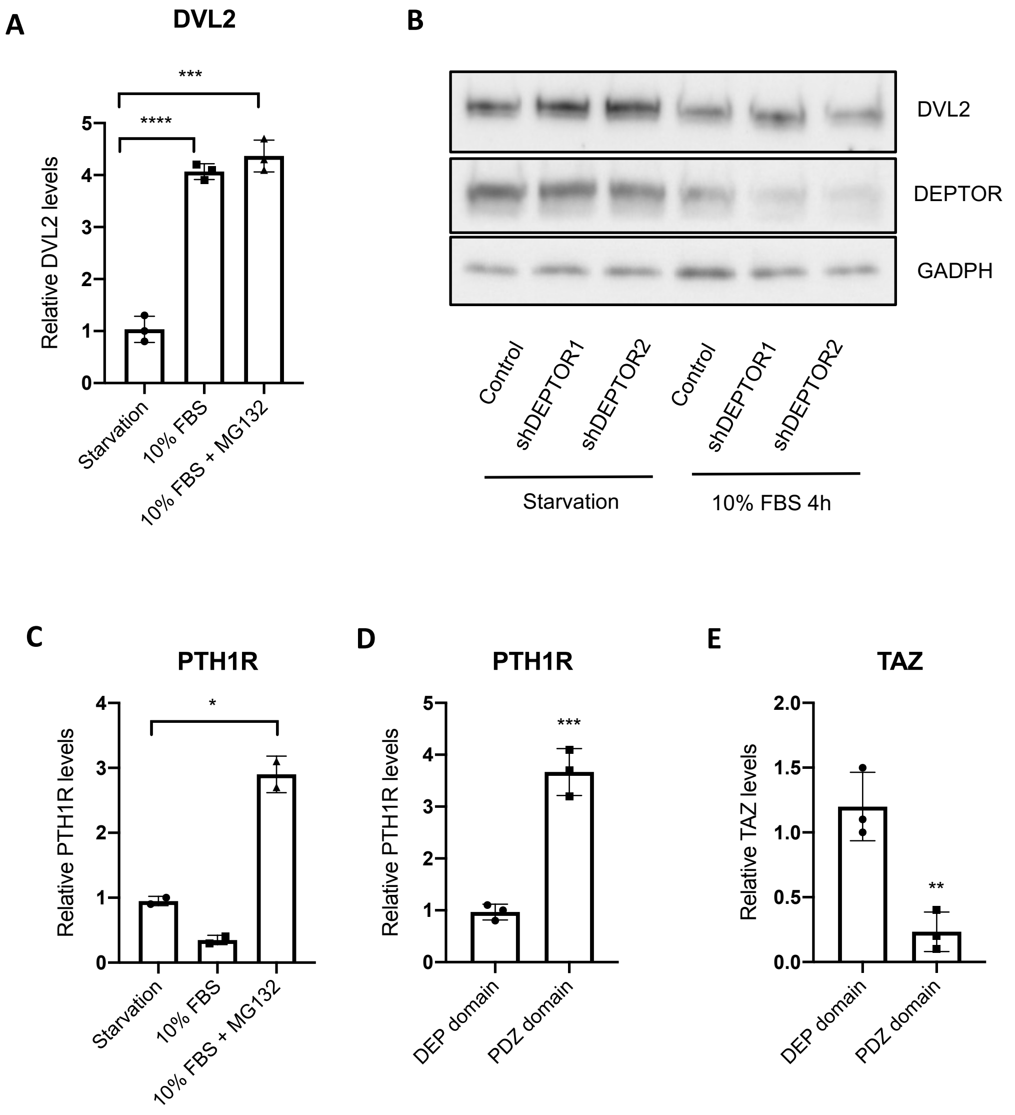
A
DVL2
Relative DVL2 levels
0
1
2
3
4
5
****
***
Starvation
10% FBS
10% FBS + MG132
B
DVL2
DEPTOR
GADPH
Control
shDEPTOR1
shDEPTOR2
Control
shDEPTOR1
shDEPTOR2
Starvation
10% FBS 4h
C
PTH1R
Relative PTH1R levels
0
1
2
3
4
*
Starvation
10% FBS
10% FBS + MG132
D
PTH1R
Relative PTH1R levels
0
1
2
3
4
5
***
DEP domain
PDZ domain
E
TAZ
Relative TAZ levels
0.0
0.5
1.0
1.5
2.0
**
DEP domain
PDZ domain

S4

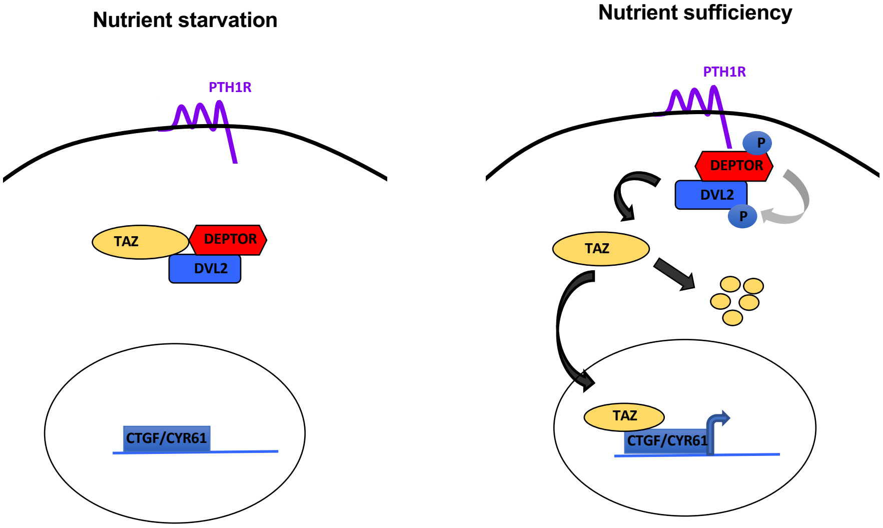

Nutrient starvation
PTH1R
TAZ
DEPTOR
DVL2
CTGF/CYR61
Nutrient sufficiency
PTH1R
P
DEPTOR
DVL2
P
TAZ
TAZ
CTGF/CYR61

Table S1. Clinical findings of affected individuals

Skeletal abnormalities	JMC	BOCD
Skeletal X Ray	Abnormal	Abnormal
Bone Density	Normal	Increased
Long Bone Size	Normal	Rhizomelia - Mesomelia - Acromelia
Spine & Vertebrae	Hypoplasia	Absent - Hypoplasia
Skull and Face	Ossification Defect	Hypoplastic - Adontia
Number Of Ribs	Decreased 11	Normal 12
Hands	Cupped - Widened	Cupped - Widened - Sclerotic
Feet	Absent Talus - Absent Calcaneous	Duplicate Calcaneous - Unusually Shaped - Short Talus
Long Bones	Widened	Shortened
